# Geographical Correlations between Indoor Radon Concentration and Risks of Lung Cancer, Non-Hodgkin’s Lymphoma, and Leukemia during 1999–2008 in Korea

**DOI:** 10.3390/ijerph14040344

**Published:** 2017-03-24

**Authors:** Mina Ha, Seung-sik Hwang, Sungchan Kang, No-Wook Park, Byung-Uck Chang, Yongjae Kim

**Affiliations:** 1 Department of Preventive Medicine, Dankook University College of Medicine, 119 Dandae-ro, Dongnam-gu, Cheonan, Chungnam 31116, Korea; 2 Department of Public Health Sciences, Graduate School of Public Health, Seoul National University, 1 Gwanak-ro, Gwanak-gu, Seoul 08826, Korea; cyberdoc@snu.ac.kr (S.H.); rjmcmc@gmail.com (S.K.); 3 Department of Geoinformatic Engineering, Inha University, 100 Inha-ro, Nam-gu, Incheon 22212, Korea; nwpark@inha.ac.kr; 4 Department of Natural Radiation Safety, Korea Institute of Nuclear Safety, 62 Gwahak-ro, Yuseong-gu, Daejeon 34142, Korea; k384cbu@kins.re.kr (B.U.C.); k337kyj@kins.re.kr (Y.K.)

**Keywords:** indoor radon, lung neoplasm, non-Hodgkin lymphoma, leukemia, child, spatial regression

## Abstract

Indoor radon is the second most important risk factor for lung cancer and may also be a risk factor for hematopoietic cancers, particularly in children and adolescents. The present study measured indoor radon concentration nationwide at 5553 points during 1989–2009 and spatially interpolated using lognormal kriging. The incidences of lung cancer, non-Hodgkin’s lymphoma (NHL), and leukemia, stratified by sex and five-year age groups in each of the 234 administrative regions in the country during 1999–2008, were obtained from the National Cancer Registry and used to calculate the standardized incidence ratios. After considering regional deprivation index values and smoking rates by sex in each region as confounding variables, the cancer risks were estimated based on Bayesian hierarchical modeling. We found that a 10 Bq/m^3^ increase in indoor radon concentration was associated with a 1% increase in the incidence of lung cancer in male and a 7% increase in NHL in female children and adolescents in Korea aged less than 20 years. Leukemia was not associated with indoor radon concentration. The increase in NHL risk among young women requires confirmation in future studies, and the radon control program should consider children and adolescents.

## 1. Introduction

Indoor radon is a major natural source of ionizing radiation exposure. Radon is a decay product of uranium, a naturally occurring element in granitic and metamorphic rocks [1]. Although humans can be exposed to radon via skin contact with radon or drinking water containing radon, the major exposure pathway is via the inhalation of radon gases in indoor air.

Kim et al. [2] first reported an annual arithmetic mean indoor radon level of 53 Bq/m^3^ in Korea, which is higher than the world average of 39 Bq/m^3^ and the highest level among East Asian countries [3]. About 8% of the surveyed households in Korea showed levels above the 300 Bq/m^3^ recommended by the International Commission for Radiation Protection. The average radon exposure was an estimated 1.65 mSv/year [4], which contributed to approximately 36% of the average annual ionizing exposure dose in the Korean population in 2007 [5]. Since 2011, the reported concentrations of measured indoor radon ranged from 102 to 124 Bq/m^3^ [6], suggesting the need for the implementation of effective residential radon control measures. 

Radon is the second most important carcinogen for lung cancer and should be controlled to a level as low as reasonably achievable because of the lack of threshold [3]. Indoor radon exposure is associated with a 16% increase in lung cancer risk (95% confidence intervals: 5%–32%) per 100 Bq/m^3^ [7,8,9], and exposure may also be associated with an increased risk of cancers in children, particularly malignancies of the hematopoietic system [10]. Because the potential radon exposure pathways include respiration, ingestion, and skin contact, the potential effect includes cancers of the lung including the trachea and bronchus, brain tumors via the olfactory nerve route, and the gastrointestinal tract including oral cancers. 

Although the Republic of Korea has reported indoor radon concentrations since 1989 [4], few studies have assessed its health effects in Korea [11]. Here, we examined the incidence and gender differences in malignant tumors—i.e., lung cancer, leukemia and non-Hodgkin’s lymphoma (NHL), in association with indoor radon exposure—using a geographical correlational method based on a Bayesian approach. 

We selected lung cancer in order to replicate the effect size in Korea compared to those in previous studies of other countries. Lung cancer is the fourth most common cancer in Korea [12]. We also examined the incidences of leukemia and NHL. Leukemia is the most common cancer in children, which is one of the most susceptible populations for ionizing radiation. The incidence of NHL has been rapidly increasing worldwide in recent years, including Korea [13,14], but few environmental risk factors have been identified.

## 2. Materials and Methods 

### 2.1. Indoor Radon Concentration 

**Figure 1:**
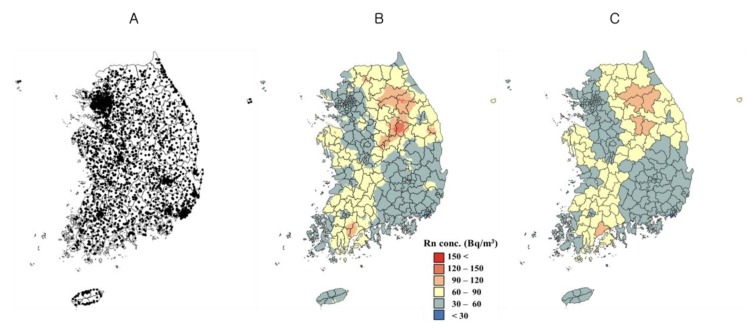
Estimated indoor radon concentrations using kriging of national radon survey data in Korea, 1989 to 2009. (**A**) Indoor radon measurement points in the national radon survey [4]; (**B**) Lognormal kriging results; (**C**) Aggregated radon concentrations in each region.

We obtained data from a national radon survey conducted four times by the Korea Institute of Nuclear Safety from 1989 to 2009, which performed a total of 5553 measurement points nationwide (Figure 1A). A detailed description has been reported elsewhere [4]. Table 1 presents a summary of the survey. 

The annual average indoor radon concentrations were used as inputs for the geostatistical interpolation. Exploratory data analysis revealed that the indoor radon concentration data sets showed a highly positively skewed distribution with a skewness value of 7.84, which means that, while most of the data values were very low, extremely large values were observed in a small number of sampling sites. Therefore, lognormal kriging was used to reduce the effects of these extreme values during spatial interpolation [15]. Ordinary lognormal kriging was implemented at 1 km grid spacing using the Geostatistical Software Library (GSLIB) [16], and an unbiased back-transform [17] was performed in Fortran in order to estimate the original indoor radon concentrations (Figure 1B). The interpolated indoor radon concentrations were finally aggregated to county-level averages for each of the 234 administrative regions using ArcGIS (ArcMap version 10.0, ESRI Inc., Redlands, CA, USA) (Figure 1C). 

### 2.2. Incidences of Lung Cancer, NHL, and Leukemia

We obtained the overall incidence rates of lung cancer (International Statistical Classification of Diseases and Related Health Problems, 10^th^ revision, ICD-10 coded C33-C34) as well as the incidence rates of NHL (ICD-10 coded C82-C85, C96) and leukemia (ICD-10 coded C90-95) overall and in children and adolescents aged 19 years or less, stratified by sex and five-year age groups in each of the 234 administrative regions (Si/Gun/Gu: the second highest level of region) from 1999 to 2008 from the National Cancer Registry [18]. 

We calculated the standardized incidence ratio (SIR) of cancers [19] by using the information on population count in 2009 from registered residents for each age group, sex, year, and administrative region as the denominator [20]. The age and sex standardized cancer incidence ratio in each administrative region was used for the correlation analysis with regional radon concentration.

### 2.3. Confounding Factors

We considered the regional deprivation index (DI) [21] as a potential confounding factor reflecting socioeconomic status on the relationship between radon exposure and lung cancers. The DI was developed based on the modified Carstairs deprivation score and the Townsend index [22]. The DI was constructed by scoring five factors—household overcrowdedness, male unemployment rate, head of the family in lower social class, home ownership, and substandard living resources—and z-standardizing the summed scores. The DI was calculated for all administrative regions using data from the 2005 National Census [20] and was used for analysis after quintile grouping.

Smoking rate by sex in each region was also considered as a confounding variable in the analysis. Information on the smoking rates in each region was obtained from the 2009 Community Health Survey [23], which is supplementary to the National Health and Nutrition Examination Survey and whose purpose is to provide health indices at the community level. The unit of community is the same as the administrative region in the present study.

### 2.4. Bayesian Hierarchical Modeling for Radon Exposure and Cancers

The present study utilized a hierarchical Bayesian model to examine the relationship between indoor radon concentration and SIR for cancer [24]. This method utilizes the local and neighboring observations and partition variation in complex statistical models of spatiotemporal association into simpler components [24,25]. Here, the conditional autoregressive model, first proposed by Besag et al. [26], was applied to model the spatial dependency between radon concentration and cancer incidence, a method which has been widely used in disease mapping [24,25]. Because there was no historical information on covariates, non-informative priors with zero means and extremely large variances were used [25].

Using WinBUGS version 1.4.3 (MRC Biostatistics Unit, Cambridge, and Imperial College School of Medicine, London, UK) with Markov Chain Monte Carlo (MCMC) methods, we generated five parallel chains, each with 20,000 samples, removed the initial 5000 iterations as the burn-in period, and systematically sampled during each of the five iterations to reduce autocorrelation in the sample [27]. In addition, the convergence of the chain was assessed by the Gelman–Rubin statistic [28]. Finally, we estimated the coefficients and calculated the relative risk and its 95% credible intervals as the post probability distribution for each cancer.

## 3. Results

Table 2 presents the characteristics of the 234 community regions. The study regions showed an average population of 172,857 individuals (range: 10,325–1,073,149). The median aggregated indoor radon concentration was 57 Bq/m^3^ with a maximum of 231 Bq/m^3^. Approximately 5.3% of the mean values of the 5553 measurement points were above the 148 Bq/m^3^ level recommended by the Korean Ministry of Environment (Ministry of Environment, Act of Indoor Air Quality Control). The average smoking rates were 50.4% and 6.4% in men and women, respectively, in 2009. During the 10-year study period, there were 115,534 and 43,178 reported cases of lung cancers, 2843 and 2051 NHL, and 15,805 and 11,696 leukemia in men and women in Korea, respectively. Among children and adolescents less than 20 years of age, there were 2843 and 2051 NHL, and 1044 and 523 leukemia cases, respectively. The maximum SIRs for cancers ranged from 1.67 to 4.99.

In male subjects, the relative risk of lung cancer per 10 Bq/m^3^ increase was 1.01, a statistically significant difference; however, the risk of NHL did not increase. In contrast, the risk of lung cancer did not show a significant increase, while a 10 Bq/m^3^ increase in indoor radon significantly increased the risk of NHL in female subject by 4%, mainly due to the high risk in children and adolescents (relative risk: 1.07, 95% credible interval: 1.01, 1.13). However, the risk of leukemia was not associated with indoor radon concentration overall or in those subjects of both sexes less than 20 years of age (Table 3).

## 4. Discussion

We found that a 10 Bq/m^3^ increase in indoor radon concentration was associated with a 1% increase in lung cancer in male and a 7% increase in NHL in female children and adolescents less than 20 years of age. However, the incidence of leukemia was not significantly associated with indoor radon concentration in all or those less than 20 years of age in Korea.

Indoor radon exposure has been associated with increased lung cancer incidence and mortality. The findings of the present study are consistent with those of previous reports. While the 1% increase in lung cancer risk was observed in both male and female subjects in the present study, the difference was significant only in males. Because smoking has a modifying effect on the association between radon exposure and lung cancer [29], this gender difference may be explained in part by the gender differences in smoking rates (50.4% and 6.0% in males and females, respectively). While smoking does not seem to be an important confounding factor for the estimation of lung cancer risk from radon exposure [30], the synergistic effect of smoke and radon on lung cancer might influence the overall estimate of lung cancer risk.

Besides the lungs, radon can also deliver a dose of alpha radiation to the bone marrow, with further impacts on the hematopoietic and lymphatic systems [10].

The risk of leukemia has been weakly associated with indoor radon exposure in children and adolescents and these results remain controversial; i.e., positive associations have been reported in most ecological studies, while null associations have been reported in most case-control studies [31,32]. We did not observe a significant association between indoor radon level and leukemia incidence in both genders among children and adolescents in the present study, which was consistent with a recent prospective analysis that showed a null association [33].

The incidence of NHL, which comprises 95.4% of lymphoma cases in Korea [34], has dramatically increased worldwide [13] and in Korea [14]. This increase cannot be explained only by increased detection rates due to advances in medical technologies. Very little is known about the environmental risk factors of NHL in early life [35,36]. Increased NHL risk in children has been associated with exposure to high levels of ionizing radiation [37], but studies in mine workers did not show a significant association between NHL and radon exposure [38,39]. To the best of our knowledge, this is the first study to show a significant NHL risk in female children and adolescents in association with indoor radon exposure. The increased risk of NHL in females was likely due to the risk in children and adolescents in the present study, which is not consistent with the previous reports that showed no clear association with NHL in children and adolescents [32,40]. However, these previous studies did not stratify their analysis by gender. 

The present finding of increased risk of NHL in female subjects is supported by those of a recent study, which showed a significant increase in the incidence of lymphoid malignancies in women living in a county with a higher radon level than in those living in a county with a lower radon level [41]. Furthermore, the study showed a comparable risk estimate of 40%/100 Bq/m^3^ [41] compared to the 4%/10 Bq/m^3^ observed in the present study. Although the gender difference should be investigated further, one possible explanation is that more women stay at home for longer periods of time compared to men [42]; thus, the measured radon level might be less misclassified in women than in men [41]. A higher baseline rate of NHL in men might be exposed to various hazardous factors due to behavioral patterns and occupational settings, which leads to the higher contribution of indoor radon to cancer risk in women [41], and this may also be true in the Korean population in the present study, in which men had a higher baseline rate of NHL [14]. 

In the present study, indoor radon measurements were performed over a long period of time (1989 to 2009); while most of the measurements were made in dwellings, some were also collected in public buildings. Presumably, the natural release of radon and its progeny does not vary over time because its source is soil granite [4]. However, the strengths of this study include the measurements over a long time period, including those in public buildings as well as in dwellings, particularly in schools, measurements across all seasons, and in various housing types and from representatively sampled sites, which combined may better represent the indoor exposure level. 

In addition, the present study used Bayesian methods for small area analysis and disease mapping, which might have improved the estimation precision by utilizing both local and neighboring observations [25]. 

This study has several limitations. First, the ecological correlational study design does not guarantee a causal association between indoor radon exposure and the risk of cancer because of the “ecological fallacy” at an individual level. Particularly for the yet unconfirmed associations of leukemia and NHL with radon exposure, the results should be interpreted carefully. However, this design can provide a community-level risk factor [43]. The ecological study results are more valuable for direct inference at the community level with respect to environmental public health interventions [44], particularly in terms of an established causal relationship, such as indoor radon and lung cancer.

Second, we could not fully adjust for the potential confounding factors in the regions. For example, there is evidence of an inverse association between NHL and ultraviolet radiation exposure [45]. However, if the unadjusted factor does not have a stronger association with the outcome than the factor of interest, the percent interpretation of the potential confounding factor for the association is not that high and the estimates will be minimally biased [46].

Third, NHL is a collective term including heterogeneous subtypes of disease and some chronic lymphocytic leukemia (CLL) can be classified as NHL. However, the incidence of CLL in Asia is far lower than that in Western countries: less than 5% versus about 30% of all leukemia, respectively [47]. Furthermore, a small portion of NHL cases (1.3%) was identified as CLL or small lymphocytic lymphoma in a Korean nationwide survey based on pathological findings in 2005 and 2006 [34]. Therefore, the degree of misclassification between NHL and leukemia might not be significant in the present study.

Finally, we could not consider the migration of people from region to region during the study period, which may contribute to the misclassification of radon exposure at the individual level. The Korean migration rate based on the unit of the study region was about 10% in 2009, which is higher than that in the US (3.7%) or in Japan (2%) at similar regional levels in 2009 [48]. If the migration is not related to radon exposure level, then it can be reasonably assumed that misclassification is not differential between different exposure groups. Under certain conditions, the non-differential exposure misclassification can skew the results toward the null [49] and lead to the underestimation of the effect size in the present study.

## 5. Conclusions

We observed an increased risk of lung cancer in men and an increased risk of NHL in women, particularly in young women, in association with indoor radon exposure in South Korea. The significantly increased risk of NHL in women requires confirmation in future prospective cohort studies. 

## Figures and Tables

**Table 1:** Summary of the national radon survey from 1989 to 2009, Korea.

Site (No. of Measurement Point)	Device	Comments	Annual Radon Concentration (Bq/m^3^)		
			Arithmetic Mean	Geometric Mean	Max.
**1st Survey (1989)**					
Dwellings (530)	RadTrack	- indoor measurements only during the winter	103.9	92.2	496
**2nd Survey (1999–2000)**					
Dwellings for three months (2953)	RadTrak	- calculation of annual average indoor concentration - distribution by season, house type, building age - focused on urban areas	52.5	42.1	1350
**3rd Survey (2002–2005)**					
Dwellings for three months (Rn 970, Tn 450)	RadTrak, Radpot, thoron progeny monitor	- indoor Rn distribution map - distribution by season, house type, building age - more rural areas	66.3	55.7	1186
**4th Survey (2008–2009)**					
Public buildings (1100)	Raduet	- 63% schools, 36.3% local governmental offices - measured on the first floor of the buildings	79.3	60.5	1004
Total (5553)			62.1	49.0	1350

This information was abstracted from a previous report by Kim et al. [4].

**Table 2:** Distribution of characteristics among 234 administrative regions in Korea.

Characteristics of the 234 Regions	Mean	Min.	Max.
Population (no. 2009) ^a^	172,857	10,325	1,073,149
Male	88,090.5	5393	539,655
Female	85,348.5	4935	533,494
Median age (years, 2009) ^a^	42.8	32.8	59.2
Male	40.8	31.5	56.1
Female	44.8	33.1	62.6
Aggregated indoor radon concentration (Bq/m^3^)	57	24	231
Deprivation index (z-score, 2005)	0.06	−7.82	7.28
Smoking rate (%, 2009) ^b^	26.7	16.4	33.4
Male	50.4	31.9	62.4
Female	6.0	0.4	12.5
Age-standardized incidence ratio ^c^			
Lung cancer			
Male (no. of cases) ^e^	1.05 (494)	0.51 (26)	1.53 (1617)
Female (no. of cases) ^e^	1.00 (185)	0.48 (11)	1.42 (736)
Non-Hodgkin’s lymphoma, all			
Male (no. of cases) ^e^	1.01 (55)	0.44 (3)	1.70 (256)
Female (no. of cases) ^e^	0.99 (43)	0.46 (3)	2.00 (211)
Non-Hodgkin’s lymphoma, children and adolescents ^d^			
Male (no. of cases) ^e^	1.00 (12)	0.00 (0)	3.93 (69)
Female (no. of cases) ^e^	0.98 (9)	0.00 (0)	3.42 (51)
Leukemia, all			
Male (no. of cases) ^e^	0.98 (68)	0.46 (4)	1.48 (283)
Female (no. of cases) ^e^	0.98 (50)	0.32 (0)	1.65 (232)
Leukemia, children and adolescents ^d^			
Male (no. of cases) ^e^	0.94 (4)	0.00 (0)	4.99 (25)
Female (no. of cases) ^e^	0.99 (2)	0.00 (0)	6.13 (14)

^a^: data from the 2009 Resident Registered Population of Korea (Korean Statistical Information System); ^b^: age-standardized smoking rate obtained from 2009 Community Health Survey data (Korea Center for Disease Control and Prevention); ^c^: standardized by five-year age group and using Central Cancer Registry data for the number of cancers from 1999 to 2008; ^d^: leukemia among people less than 20 years of age; ^e^: total number of cancer cases, 1999–2008.

**Table 3:** Relative risk and 95% credible intervals for lung cancer, non-Hodgkin’s lymphoma, and leukemia in relation to regional indoor radon concentrations in 234 regions of Korea, 1999 to 2013.

	Per 10 Bq/m^3^ Increase in Radon Concentration	Crude		Adjusted ^a^	
	Cancer Type	Relative Risk	95% Credible Intervals	Relative Risk	95% Credible Intervals
Male	Lung cancer	1.03	(1.02, 1.05)	1.01	(1.00, 1.02)
	NHL, all	1.01	(0.99, 1.03)	1.00	(0.98, 1.02)
	NHL in children and adolescents ^b^	0.98	(0.94, 1.02)	0.97	(0.93, 1.02)
	Leukemia, all	0.98	(0.96, 1.00)	0.98	(0.96, 1.00)
	Leukemia in children and adolescents ^b^	0.96	(0.89, 1.03)	1.00	(0.92, 1.08)
Female	Lung cancer	1.01	(0.99, 1.02)	1.01	(0.99, 1.02)
	NHL, all	1.03	(1.01, 1.06)	1.04	(1.02, 1.07)
	NHL in children and adolescents ^b^	1.04	(0.99, 1.10)	1.07	(1.01, 1.13)
	Leukemia, all	0.98	(0.96, 1.01)	0.98	(0.95, 1.00)
	Leukemia in children and adolescents ^b^	1.00	(0.91, 1.10)	0.98	(0.88, 1.08)

Relative risk and 95% credible intervals estimated using the hierarchical Bayesian model with Markov Chain Monte Carlo methods; ^a^: adjusted for smoking rate and regional deprivation index; ^b^: less than 20 years of age; NHL: non-Hodgkin’s Lymphoma.
